# How social support impacts depression in Chinese emerging adults: mediators of cognitive reappraisal and loneliness

**DOI:** 10.3389/fpsyg.2026.1792698

**Published:** 2026-03-19

**Authors:** Fangyan Lv, Cheng Lv, Burebiya Abudurexiti, Qingyuan Ma, Qingxi Yang, Minhui Ouyang, Dongzhe Shi

**Affiliations:** 1 School of Marxism, Sun Yat-sen University, Guangzhou, China; 2 School of Forestry and Landscape Architecture, Anhui Agricultural University, Hefei, China; 3 Department of Psychiatry, The First People’s Hospital of Kashi, Kashi, China; 4 Boya (Liberal Arts) College, Sun Yat-sen University, Guangzhou, China; 5 School of Information Management, Sun Yat-sen University, Guangzhou, China; 6 Department of Sports Science and Physical Education, Guangzhou Xinhua University, Guangzhou, China

**Keywords:** Chinese emerging adults, cognitive reappraisal, depression, expressive suppression, social support

## Abstract

**Background:**

Emerging adulthood marks a crucial maturation from teenage years to independent adulthood, which makes them more susceptible to depression. Prior studies have indicated that social support negatively impacts depression among emerging adults. However, little research has explored the mediating mechanisms.

**Methods:**

This research investigated the potential effectiveness of social support in mitigating depression through emotion regulation strategies and loneliness as mediators in 998 college students (M = 19.18, SD = 1.99). Data analyses were conducted using SPSS version 23.0 and PROCESS version 3.3. Both correlational and mediation analyses were performed to examine the study’s hypotheses.

**Results:**

Correlational analyses revealed that PSS was negatively associated with both loneliness and depression, and positively associated with cognitive reappraisal. Mediation analysis indicated that the direct effect of PSS on depression became non-significant (*β* = −0.06, *p* > 0.05) after accounting for mediating variables, confirming a full mediation model. The final model explained 47% of the variance in depression (*R^2^* = 0.47, *F* = 148.21, *p* < 0.001). Bootstrap analysis identified three significant indirect pathways: (1) *PSS → cognitive reappraisal → depression* [indirect effect = 0.13, 95% CI (−0.17, −0.09)]; (2) *PSS → loneliness → depression* [indirect effect = 0.16, 95% CI (−0.21, −0.12)]; and (3) the serial pathway *PSS → cognitive reappraisal → loneliness → depression* [indirect effect = 0.04, 95% CI (−0.07, −0.02)]. The pathway through loneliness exhibited the strongest indirect effect. These further demonstrated that the overall impact of perceived social support on depression was fully mediated by these two variables because expressive suppression did not appear as a substantial mediator.

**Conclusion:**

The findings suggest that perceived social support reduces depressive symptoms primarily by facilitating the use of cognitive reappraisal and alleviating loneliness. These results underscore the critical role of social support systems in mental health and highlight the importance of fostering adaptive emotion regulation strategies. Such initiatives are promising for buffering loneliness, mitigating depressive risk, and promoting the psychological well-being of emerging adults.

## 1 Introduction

Depression presents itself as a worldwide psychological health problem, which threatens mental health, particularly during the emerging adulthood transitional period of phase emerging adulthood (Arnett, 2007). The life stage of emerging adulthood (ages of 18–29) is a period of changes and challenges, during which people are more vulnerable to mental health problems (Arnett, 2007) and psychopathological conditions (Bernstein et al., 2021), including feelings of loneliness (Kirwan et al., 2024) and depressive symptoms (Yichen and Chuntian, 2024). Emerging adults are susceptible to depression due to identity uncertainty, academic and career pressure, financial burden, alteration in interpersonal relationships, influence of technology, and lack of social support (Osma et al., 2022). Inadequate social support to cope with these challenges may increase the risk of depressive symptoms (Lakey and Cronin, 2008). Recent data in China show that 14.8% of adolescents exhibit depressive risk, and emerging adults face unique stressors like urbanization, education competition, and digital era social isolation (National Health Commission of China, 2024; Institute of Psychology, Chinese Academy of Sciences, 2021). In light of these challenges, social support proves to be an important protective factor in reducing the mental health risks in this period of development. A body of empirical work has shown that social support and depression are inversely related; higher levels of social support are associated with a lower prevalence of depressive symptoms (Mitchell et al., 2025). Additionally, loneliness has been linked to a lack of social support (Lv et al., 2022). However, the pathways through which social support confers its protective effects have not been fully characterized in emerging adulthood.

To theoretically ground the present study, we integrate two complementary frameworks: Social Support Theory (Cohen and Wills, 1985) and the Cognitive-Behavioral Theory (CBT) of Depression (Beck, 1967). As a macro-level framework, Social Support Theory posits that social support acts as a critical protective factor against depression by providing individuals with tangible resources, emotional validation, and coping guidance, distinguishing between direct and buffering effects, both relevant to emerging adults’ mental health. Complementing this at the micro-level, CBT emphasizes that depressive symptoms stem from interactions between negative cognitive patterns, maladaptive emotion regulation, and adverse emotional experiences, highlighting cognitive reappraisal and loneliness as key mechanisms linking external factors to depressive outcomes. Together, these two theories form a cohesive theoretical foundation for our research model: Social Support Theory explains the predictive role of social support in depression, while CBT clarifies why cognitive reappraisal and loneliness serve as mediators, as well as their sequential relationship.

### 1.1 Social support and depression

Social support, conceptualized as the perceived or actual assistance provided by social networks, has proven to be a critical buffer in combating depression. Extensive studies have indicated that social support can alleviate the negative influence of stress on mental health and reduce the potential for depression (Cohen and Wills, 1985; Cohen et al., 2000; Fang et al., 2021; Lakey and Cronin, 2008; Rueger et al., 2016; Wang et al., 2018; Mohd et al., 2019). Systematic reviews in Asian communities further confirm its universal protective role (Sun et al., 2023; Cai and Wu, 2024), while longitudinal studies highlight its enduring benefits for mental health (Yuan et al., 2024). This buffering effect has been consistently confirmed across diverse populations, including older adults (Liu et al., 2016; Hao et al., 2023), clinical populations such as caregivers of cancer patients (Nuwamanya et al., 2023; Schiller et al., 2021), women with breast cancer (Zamanian et al., 2021), and individuals living with HIV/AIDS (Sun et al., 2023). For adolescents and young adults, social support during adolescence significantly lowers risks of depression, anxiety, and suicidal ideation (Ma et al., 2023; Seet et al., 2024), with perceived support from peers and family acting as a buffer against academic and social stressors (Alsubaie et al., 2019; Sadeghi et al., 2023). Notably, in migrant populations, perceived social support directly relates to reduced depression and anxiety, emphasizing its universal relevance (Ma et al., 2024). The COVID-19 pandemic emphasizes social support’s function in mitigating depression, particularly among isolated young adults, where adaptive coping strategies mediate this relationship (Narita et al., 2023; Zhao et al., 2022).

Despite the well-documented protective function of social support against depression conducted in diverse populations, the mechanisms by which it operates are not yet completely clear. The buffering hypothesis (Cohen and Wills, 1985) suggests that social support moderates the negative correlation between stress and mental health by providing individuals with coping resources. Empirical research has revealed that people with robust social support networks are less inclined to develop depression, even when exposed to significant stressors (Cohen et al., 2000; Lakey and Cronin, 2008). Social support can enhance self-esteem, foster belonging, and reduce loneliness, significantly mitigating depressive symptoms (Turner and Brown, 2010). A meta-analysis further indicated a notable link between perceived social support and depression across diverse populations, highlighting its universal protective role (Wang et al., 2018).

However, the pathways by which social support influences depression may vary across developmental stages and cultural contexts. In collectivist cultures, such as those in China, social support is often deeply embedded in familial and community networks, and the loss or absence of such support may profoundly affect mental health (Wei et al., 2005). Emerging adults in China who have less family support are more likely to experience depression (Sun et al., 2017). This emphasizes the significance of considering cultural factors when examining the protective role of social support.

Given the complexity of the link between social support and depression, further investigation is required to clarify the specific mechanisms by which social support offers its protective benefits, particularly during emerging adulthood and across different cultural contexts. However, the pathway through which social support plays a protective role is not fully understood. The study endeavors to fill this gap by investigating how social support alleviates depression and uncovering the potential mechanisms. Therefore, the first hypothesis that needs to be verified is whether social support has a significant negative impact on depression in Chinese emerging adults.

Having established the link between social support and depression, research has subsequently paid attention to elucidating the specific mechanisms that drive this connection. Mediation analysis has become a prevalent method in these investigations. Although various subjective psychological factors have been acknowledged as mediators impacting the link between social support and depression, these factors alone are insufficient to fully elucidate the underlying mechanism. For example, constructs such as stigma (Wang et al., 2022), self-control (Zhao et al., 2022), self-efficacy (Zhang and Jin, 2016), resilience (Sun et al., 2023), and lifestyle (Ma et al., 2024) have been suggested as potential mediators. Nonetheless, these variables do not collectively capture the intricate pathways through which social support influences depression. In this research, loneliness and emotion regulation strategies are the main mediating variables under investigation.

### 1.2 Loneliness as a mediator

Social support has a direct influence on depression and may also influence depression through individuals’ subjective psychological factors. Loneliness has been determined to be a critical mediator in the link between social support and mental health (Hawkley and Cacioppo, 2010). Prior research has indicated that there is a correlation between inadequate social support and depression (Gariepy et al., 2016; Luo et al., 2025), but they have not yet explored the experience of loneliness. A significant research gap is evident, considering that loneliness is established as an independent factor influencing depression (Ma, 2020; Mann et al., 2022).

Individuals in emerging adulthood, who are transitioning from adolescence to full-fledged adulthood, often experience fluctuations in their social connections due to life transitions like moving out, entering college, or beginning a career (Arnett, 2000; Kirwan et al., 2024). These changes can disrupt existing social ties and create challenges in forming new relationships, leading to heightened feelings of loneliness (Matthews et al., 2016; Vanhalst et al., 2015). For instance, studies have shown that college students who move away from their hometowns often struggle with the loss of familiar social support systems, which can intensify feelings of isolation and disconnection (Buote et al., 2007). Therefore, emerging adulthood loneliness has been significantly related to indicators of poorer mental health (Kirwan et al., 2024). Consistent with the integrated theoretical framework, loneliness is positioned as a key mediator between social support and depression, jointly supported by Social Support Theory and CBT. From the perspective of Social Support Theory (Cohen and Wills, 1985), social support directly alleviates loneliness by fulfilling unmet social connection needs, thereby reducing depressive risks indirectly. From a CBT standpoint (Beck, 1967), loneliness, as a negative emotional experience, reinforces negative cognitive biases, which in turn perpetuate depressive symptoms.

Loneliness has been consistently related to increased depression (Cacioppo et al., 2010; Hawkley and Cacioppo, 2010; de la Fuente et al., 2018). In a longitudinal study, loneliness in emerging adulthood was linked to increased depression levels over time, independent of initial mental health status (Matthews et al., 2016). In addition, loneliness mediates the correlation between social support deficits and depression, suggesting that the absence of strong social connections can induce feelings of isolation, which then help bring about or worsen depression (Vanhalst et al., 2015). Thus, loneliness may serve as a key mechanism by which low social support contributes to depression, particularly in the absence of strong social connections. When individuals lack social support, they are more inclined to experience loneliness, which can trigger maladaptive cognitive and emotional processes (Cacioppo et al., 2010). These processes, in turn, increase vulnerability to depressive symptoms. Furthermore, loneliness can create a feedback loop, where depressive symptoms further isolate individuals from potential sources of social support, perpetuating a cycle of loneliness and depression (Hawkley and Cacioppo, 2010).

Loneliness has been established as a vital mediator in the link between social support and depression across various populations and contexts. Studies have consistently shown that perceived social support can mitigate loneliness, which in turn reduces the chance of developing depression (Bareket-Bojmel et al., 2021; Chen et al., 2024; Jensen-Campbell et al., 2023; Liu et al., 2022; Liang et al., 2019; Roskoschinski et al., 2023). Individuals with depression symptoms often experience stigma, which reduces social support and increases loneliness, further exacerbating their mental health challenges (Prizeman et al., 2023). Among men who have sex with men, lower social support and higher loneliness were linked to greater depression, highlighting the intersection of social isolation and minority stress (Xie et al., 2024). Similarly, individuals with perfectionistic tendencies or social disconnection exhibit amplified loneliness due to perceived inadequacy in meeting social expectations, which exacerbates depressive outcomes (Chen et al., 2024). These findings stress the vital role of loneliness as a pathway by which social support affects mental health. By addressing loneliness as a critical pathway linking social support to depression, this research hypothesized that loneliness may play a mediated role in the link between social support and depression in emerging Chinese adults.

### 1.3 Emotion regulation strategies as a mediator

In addition to loneliness, emotion regulation strategies have been considered as another key mechanism linking social support to depressive symptoms. Emotion regulation, characterized as the processes involved in managing emotional experiences (Gross, 1998), plays a pivotal role in determining how people respond to stressors and social interactions. Cognitive reappraisal is strongly associated with psychological resilience and depression (Gross and John, 2003; Aldao et al., 2010). For example, individuals who employ cognitive reappraisal tend to reassess stressful events in ways that align with long-term goals, thereby mitigating negative emotional outcomes (Troy et al., 2013). By contrast, maladaptive strategies like expressive suppression are linked to heightened depressive symptoms, as they prevent emotional processing and exacerbate internal distress (Joormann and Stanton, 2016; Nolen-Hoeksema et al., 2008). Studies have revealed that habitual use of suppression correlates with increased rumination, social disengagement, and impaired interpersonal functioning, all of which elevate depression risk (Hofmann et al., 2009; English et al., 2012). Within our integrated theoretical framework, cognitive reappraisal is a critical mediating mechanism, aligned with both Social Support Theory and CBT. CBT (Beck, 1967) identifies cognitive reappraisal as a core strategy to reframe negative thoughts, thereby mitigating depressive symptoms—consistent with our focus on its adaptive role. Social Support Theory (Cohen and Wills, 1985) further complements this by suggesting that social support fosters the use of cognitive reappraisal: supportive relationships provide a safe space for individuals to receive alternative perspectives, enabling them to reframe stressors adaptively.

Social support may be a catalyst for adaptive emotion regulation by fostering a sense of security and validation. When people feel strong social support, they tend to engage in cognitive reappraisal, as supportive relationships provide a safe space to reframe challenges and receive constructive feedback (Marroquín, 2011). For instance, supportive peers or family members may model adaptive coping strategies or offer alternative perspectives that encourage reappraisal (Hofmann et al., 2016). Conversely, inefficient social support may be related to the utilization of maladaptive strategies, like suppression, which can exacerbate depressive symptoms (Hofmann et al., 2009). Without external validation or coping resources, individuals may internalize negative emotions, leading to a cycle of emotional avoidance and heightened depression (Nolen-Hoeksema et al., 2008). This dynamic is particularly salient in collectivist cultures like China, where social harmony and familial expectations may amplify pressure to suppress emotions to avoid burdening others (Wei et al., 2005). For instance, Chinese emerging adults who prioritize familial obligations may suppress personal distress to maintain group cohesion, inadvertently worsening depressive outcomes (Chen et al., 2009). Thus, emotion regulation strategies serve as both a bridge and a barrier in the correlation between social support and depression. By elucidating how social support shapes the adoption of adaptive or maladaptive strategies, this framework underscores the importance of interpersonal resources in fostering emotional resilience during emerging adulthood.

### 1.4 The sequential mediated effect of emotion regulation strategies and loneliness

Emotion regulation strategies and loneliness are also interrelated. Empirical studies have supported that there is a link between emotion regulation strategies and loneliness. Now the direction of the influences is not clear. Because loneliness and emotion regulation strategies may impact each other (Patrichi et al., 2024). Studies have revealed that cognitive reappraisal was negatively correlated to loneliness (Dawel et al., 2021), and expressive suppression was positively related to loneliness (Patrichi et al., 2024). Thus, given the prior discussion, it seems plausible that social support may also affect depression through the sequential mediation of emotion regulation strategies and loneliness.

### 1.5 Present study

**Figure 1:**
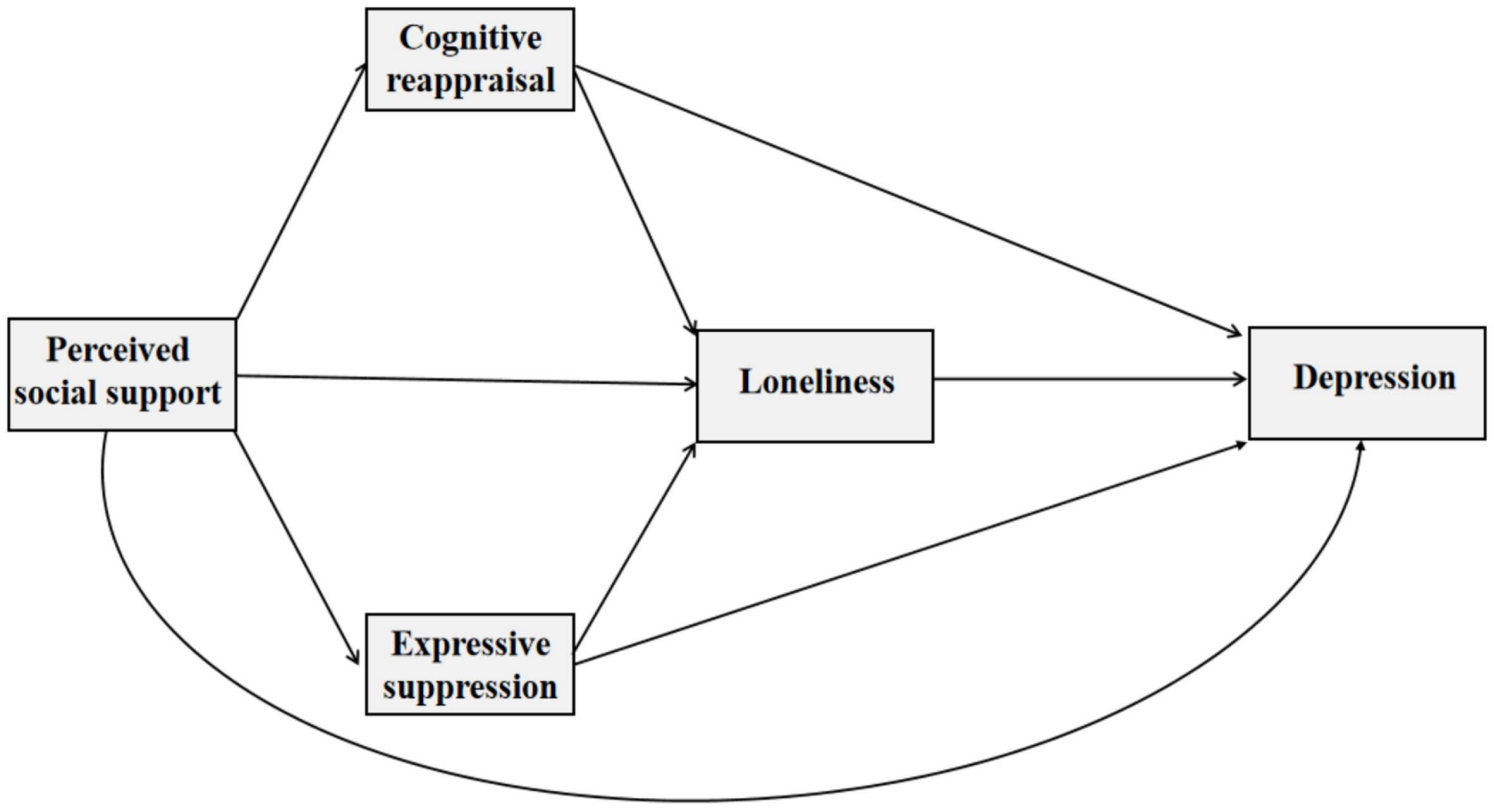
The proposed chain mediation model.

As highlighted earlier, a large body of empirical evidence has explored the factors influencing depression among emerging adults, particularly focusing on the links between depression and social support, loneliness, and emotion regulation strategies. However, a significant gap still exists in the literature, as little research has explored the interrelationships among these factors, especially in the context of Chinese emerging adults. This research, therefore, seeks to deal with this question by exploring the underlying mechanisms through which social support affects depression in Chinese emerging adults, with a particular focus on mediators of loneliness and emotion regulation strategies. The following research hypotheses were established (see Figure 1):

H1. Social support has a significant negative influence on depression in Chinese emerging adults.

H2. Social support is negatively linked with loneliness in Chinese emerging adults.

H3. Social support is positively correlated with cognitive reappraisal, but not significantly related to expressive suppression among Chinese emerging adults.

H4. Cognitive reappraisal mediates the correlation between social support and depression in Chinese emerging adults.

H5. Expressive suppression does not mediate the correlation between social support and depression in Chinese emerging adults.

H6. Loneliness mediates the link between social support and depression in Chinese emerging adults.

H7. Cognitive reappraisal and loneliness are chain mediators in the link between social support and depressive symptoms among Chinese emerging adults.

H8. Expressive suppression and loneliness do not act as chain mediators in the link between social support and depressive symptoms in Chinese emerging adults.

## 2 Method

### 2.1 Participants and procedure

This research conducted convenience sampling to select participants. Between February and March 2024, a random sample of 1,020 young Chinese adults was recruited from two universities in Guangzhou, Guangdong Province, South China, using Wenjuanxing. Responses were deemed invalid and omitted if they included continuous high-frequency fixed options (Curran, 2016), lie test errors (DeSimone et al., 2015), or if response times were less than 1 min, resulting in 998 valid responses for analysis (response effectiveness rate = 97.84%). A total of 998 valid participants were included, comprising 496 females and 502 males, and participants’ ages extended from 18 to 33 years (M = 19.18, SD = 1.99). All participants finished their written consent after being fully informed. Course credit was given to each participant as compensation. The research involving humans has been ethically approved by the first author’s Institutional Review Board.

### 2.2 Measures

#### 2.2.1 Perceived social support

The Multidimensional Scale of Perceived Social Support (MSPSS, Zimet et al., 1988) was employed in this study. This scale comprises 12 items, including three aspects: support from others, family support, and friend support. A 7-point scale was used, with responses ranging from “Strongly Disagree” (1) to “Strongly Agree” (7). An increased total score suggests greater perceived overall social support. This scale’s alpha coefficient was 0.94.

#### 2.2.2 Emotion regulation

The 10-item Emotion Regulation Questionnaire (ERQ; Gross and John, 2003) was utilized to evaluate the general propensities of participants to employ two distinct strategies: cognitive reappraisal (for instance, “I managed my emotions by altering my perspective on the situation I am facing”) and expressive suppression (for example, “I hold my emotions inside, I do not let others see what am feeling”; Gross and John, 2003). Items were evaluated on a 7-point Likert scale, following 1 for “strongly disagree” and 7 for “strongly agree.” A Cronbach’s *α* coefficient of 0.93 was observed for this ERQ in the present research.

#### 2.2.3 Loneliness

Feelings of loneliness were assessed using the Chinese adaptation of the abbreviated version of the University of California, Los Angeles Loneliness Scale (UCLS-8), an eight-item questionnaire. Each question was evaluated using a 4-point scale, where the options were 1 for “never” and 4 for “always.” The higher the scores, the greater the loneliness experienced. This scale shows a Cronbach’s *α* coefficient of 0.75.

#### 2.2.4 Depression

Depression was evaluated by the 20-item Self-Rating Depression Scale (SDS), which has shown strong psychometric properties in prior research (Beard et al., 2016; Martin et al., 2006). Participants reflected on their experiences over the last fortnight and rated the extent to which they were troubled by issues such as “I feel downhearted and gloomy” on a 4-point Likert scale, where 1 for “never” and 4 for “always.” The higher the scores, the greater the depression experienced. The strong internal reliability of the scale was supported by a Cronbach’s alpha of 0.81.

### 2.3 Data analysis

In the present research, data integrity was robust, with only minimal missing values across participants, thus eliminating the need for data deletion. All statistical analyses were performed using SPSS 23.0, with statistical significance defined at a two-tailed α level of 0.05. The analytical workflow began with an assessment of common method bias (CMB) to address potential methodological confounding. Subsequent steps included the calculation of descriptive statistics and bivariate Pearson correlations to characterize the distribution of study variables and their preliminary associations.

Building on these preliminary analyses and the study’s hypothesized framework, linear regression models were constructed to examine the relationship between physical activity and subjective well-being. To rigorously test the proposed mediation mechanisms, the PROCESS macro (Version 3.3) for SPSS was utilized (Hayes, 2018), paired with a nonparametric bootstrap procedure involving 5,000 resamples. Mediation effects were considered statistically significant if the 95% bias-corrected confidence interval (CI) did not include zero, an approach that enhances the reliability of inferences regarding the hypothesized pathways. Collectively, this analytical strategy ensures methodological rigor, reduces potential biases, and facilitates clear interpretability of the study results.

## 3 Results

### 3.1 Common method bias test

Harman’s one-factor test was employed to assess the potential presence of common method bias (CMB) in the data. Specifically, exploratory factor analysis (EFA) was conducted via SPSS to extract the first unrotated factor from the full data matrix. Results revealed that this single factor accounted for 19.67% of the total variance—well below the 40% threshold typically used to indicate that CMB dominates the variance explanation. Thus, Harman’s one-factor test suggests that CMB is unlikely to have biased the study results (Zhou and Long, 2004), confirming no significant CMB in the dataset.

### 3.2 Descriptive statistics and correlation analysis

Table 1 presents the descriptive statistics and bivariate Pearson correlations for all study variables. The sample (*N* = 998) had a mean age of 19.18 years (SD = 1.99). Depressive symptoms had a mean score of 51.47 (SD = 10.72), while PSS had a mean score of 60.44 (SD = 12.84). Consistent with hypotheses, PSS was significantly negatively correlated with depression (*r* = −0.39, *p* < 0.001) and loneliness (*r* = −0.40, *p* < 0.001), and positively correlated with cognitive reappraisal (*r* = 0.59, *p* < 0.001). Cognitive reappraisal was negatively correlated with both depression (*r* = −0.36, *p* < 0.001) and loneliness (*r* = −0.26, *p* < 0.001), whereas expressive suppression (ES) was positively correlated with depression (*r* = 0.26, *p* < 0.001) and loneliness (*r* = 0.30, *p* < 0.001). Loneliness exhibited the strongest positive correlation with depression (*r* = 0.64, *p* < 0.001), indicating a robust association between these two variables. Age and gender showed only weak or non-significant correlations with the primary study variables. As anticipated, perceived social support and cognitive reappraisal were negatively correlated with depression. In contrast, expressive suppression was positively related to loneliness and depression. Additionally, perceived social support appeared positively related to cognitive reappraisal, although it did not exhibit a significant association with expressive suppression.

**Table 1:** Descriptive statistics and correlations of depression, pss components, emotion regulation strategies, and loneliness (*N* = 998).

Variable Name	M ± SD	1	2	3	4	5	6	7
1 Depression	51.47 ± 10.72	_						
2 PSS	60.44 ± 12.84	−0.39^***^	_					
3 CR	31.24 ± 5.85	−0.36^***^	0.59^***^	_				
4 ES	16.26 ± 5.00	0.26^***^	−0.01	0.19^***^	_			
5 Loneliness	20.07 ± 4.97	0.64^***^	−0.40^***^	−0.26^***^	0.30^***^	_		
6. Age	19.18 ± 1.99	−0.04	0.05	0.02	−0.03	−0.08^*^	_	
7. Gender	—	0.02	0.03	0.02	−0.17^***^	0.02	0.04	_

PSS, perceived social support, CR, cognitive reappraisal, ES, expressive suppression.

^**^p < 0.05, ^*^p < 0.01, ^***^p < 0.001.

### 3.3 Mediation analysis

Table 2 summarizes the results of the hierarchical multiple regression analyses testing the hypothesized mediation model. In the initial model (Model 1), PSS was a significant negative predictor of depression (*β* = −0.39, *t* = −13.39, *p* < 0.001), accounting for 15% of the variance in depressive symptoms [*R*^2^ = 0.15, *F*_(3, 994)_ = 60.68, *p* < 0.001]. Age and gender did not significantly predict depression. PSS significantly positively predicted cognitive reappraisal (*β* = 0.59, *t* = −0.32, *p* < 0.001; Model 2), explaining 35% of its variance [*R*^2^ = 0.35, *F*_(3, 994)_ = 176.93, *p* < 0.001]. By contrast, PSS did not significantly predict expressive suppression (*β* = 0.002, *t* = −0.06, ns; Model 3). PSS (*β* = −0.32, *t* = −9.40, *p* < 0.001), cognitive reappraisal (*β* = −0.14, *t* = −4.04, *p* < 0.001), and expressive suppression (*β* = 0.33, *t* = 11.74, *p* < 0.001) were all significant predictors of loneliness. Together, these variables explained 27% of the variance in loneliness [*R*^2^ = 0.27, *F*_(4, 993)_ = 73.45, *p* < 0.001, Model 4]. When all mediators were entered into the model (Model 5), loneliness emerged as the strongest positive predictor of depression (*β* = 0.51, *t* = 19.03, *p* < 0.001). Cognitive reappraisal (*β* = −0.22, *t* = −7.42, *p* < 0.001) and expressive suppression (*β* = 0.16, *t* = 6.30, *p* < 0.001) also exerted significant direct effects on depression. Critically, the direct effect of PSS on depression became non-significant (*β* = −0.06, *t* = −1.83, ns), indicating a full mediation effect. The final model explained 47% of the variance in depression [*R*^2^ = 0.47, *F*_(5, 992)_ = 148.21, *p* < 0.001].

**Table 2:** Multiple regression of the mediation effect (*N* = 998).

Predictors	Model 1 (depression)		Model 2 (CR)		Model 3 (ES)		Model 4 (LS)		Model 5 (depression)	
	*β*	*t*	*β*	*t*	*β*	*t*	*β*	*t*	*β*	*t*
Age	−0.02	−0.80	−0.01	23.01	−0.03	−0.86	−0.05	−2.00	0.01	0.48
Gender	0.04	1.21	0.002	0.06	−0.17	−5.33	0.09	3.24	0.05	1.93
PSS	−0.39^***^	−13.39	0.59^***^	−0.32	0.002	−0.06	−0.32^***^	−9.40	−0.06	−1.83
CR							−0.14^***^	−4.04	−0.22^***^	−7.42
ES							0.33^***^	11.74	0.16^***^	6.30
Loneliness									0.51^***^	19.03
*R*	0.39		0.59		0.17		0.52		0.69	
*R*^2^	0.15		0.35		0.03		0.27		0.47	
*F*	60.68^***^		176.93.73^***^		9.86		73.45^***^		148.21^***^	

PSS, perceived social support, CR, cognitive reappraisal, ES, expressive suppression.

^**^p < 0.05, ^*^p < 0.01, ^***^p < 0.001.

**Figure 2:**
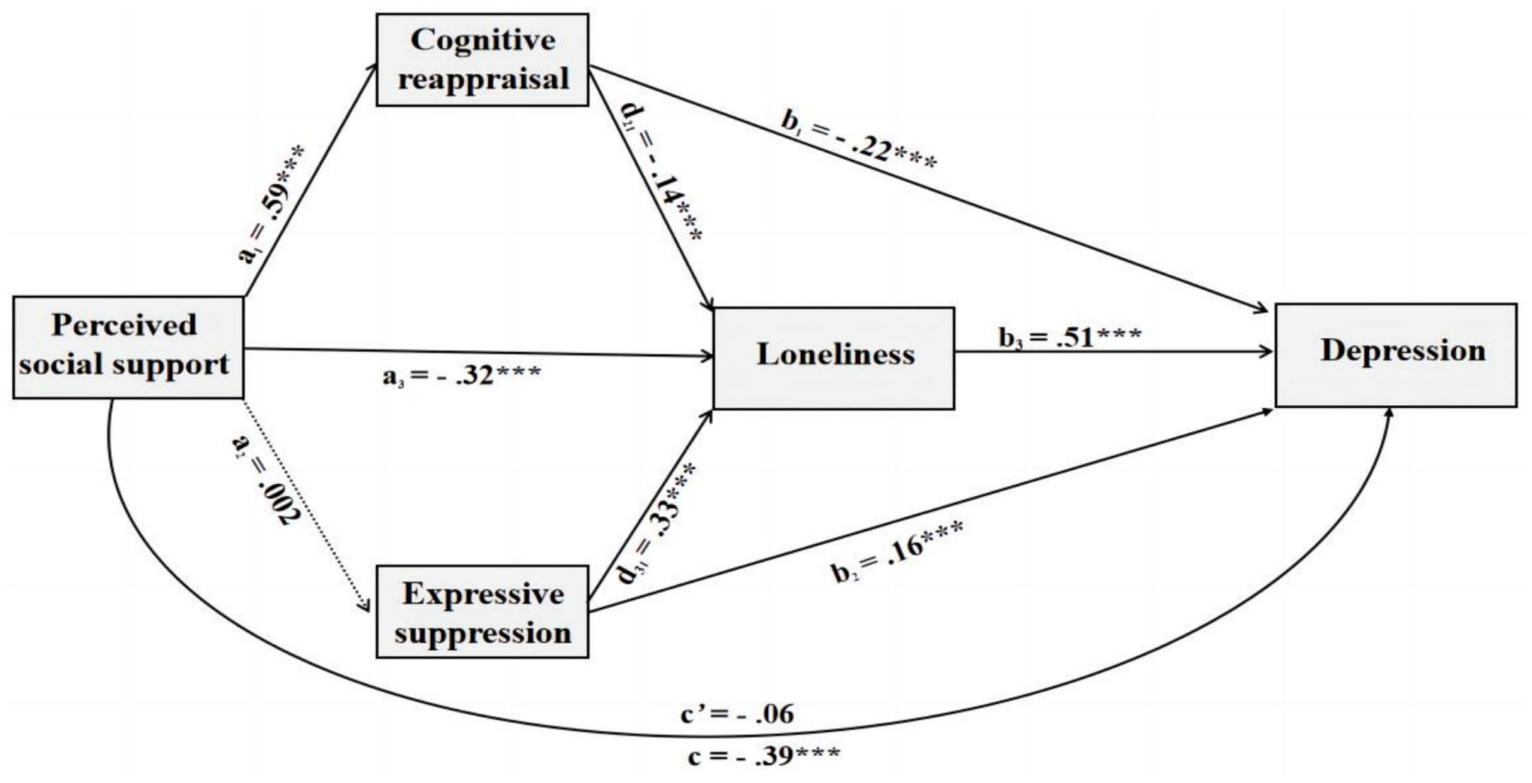
Multiple mediation model of the impacts of perceived social support on depression among Chinese emerging adults. ^**^p < 0.05, ^*^p < 0.01, ^***^p > *p* < 0.001.

Decomposition of the mediation effects (Table 3) confirmed a full mediation pattern: the total effect of perceived social support (PSS) on depression was significant [Effect = 0.33, SE = 0.02, 95% CI (−0.37, −0.28)], whereas the direct effect of PSS on depression was non-significant [Effect = 0.05, SE = 0.03, 95% CI (−0.10, 0.004)]. Three significant indirect pathways were identified: (a) PSS → cognitive reappraisal (CR) → depression [indirect effect = 0.13, SE = 0.02, 95% CI (−0.17, −0.09)]; (b) PSS → loneliness → depression [indirect effect = 0.16, SE = 0.02, 95% CI (−0.21, −0.12)]; and (c) the serial pathway PSS → CR → loneliness → depression [indirect effect = 0.04, SE = 0.01, 95% CI (−0.07, −0.02)]. Notably, the largest indirect effect was observed through the PSS → loneliness → depression pathway, highlighting loneliness as a primary mechanism through which perceived social support alleviates depressive symptoms. The overall impact of perceived social support on depression was fully mediated by these two variables because expressive suppression did not appear as a substantial mediator (see Figure 2).

**Table 3:** Decomposition of the effect of perceived social support on depression.

Effect	Path	Effect	SE	95% Boot CI
Direct effect	PSS → depression	0.05	0.03	[−0.10 0.004]
Indirect effect	PSS → CR → depression	0.13	0.02	[−0.17–0.09]
Indirect effect	PSS → LS → depression	0.16	0.02	[−0.21–0.12]
Indirect effect	PSS → CR → LS → depression	0.04	0.01	[−0.07–0.02]
Total effect	PSS → depression	0.33	0.02	[−0.37–0.28]

PSS, perceived social support, CR, cognitive reappraisal, ES, expressive suppression.

## 4 Discussion

This research aimed to explore the potential mediated effects of emotion regulation strategies and loneliness on the correlation between perceived social support and depression among Chinese emerging adults. The results of the present research suggested that depression was negatively associated with perceived social support and cognitive reappraisal, whereas loneliness positively predicted depression. However, expressive suppression was not associated with depression. The findings also revealed that cognitive reappraisal and loneliness acted as mediators in the influence of perceived social support on depression, whereas expressive suppression was not a mediator. Overall, these findings broaden our comprehension of the impact of perceived social support on depression and contribute to studies that tie depression with loneliness and cognitive reappraisal.

First, our findings offered further evidence confirming that perceived social support is a significant buffer factor for Chinese emerging adults’ depression. This result matches the majority of previous research across diverse populations (Alsubaie et al., 2019; Ma et al., 2024; Nuwamanya et al., 2023; Yang et al., 2023). Emerging adults, who are navigating a vital developmental stage typified by identity exploration, academic pressures, and shifting social dynamics, are especially susceptible to mental health challenges (Arnett, 2024). During this transitional period, perceived social support acts as a buffer against stressors by fostering a sense of belonging, enhancing self-esteem, and providing practical resources to cope with adversity (Wang et al., 2018). Notably, the protective role of perceived social support may be amplified in collectivist cultures like China, where familial and community ties are deeply ingrained (Ma et al., 2023). Perceived social support emerged as a key resilience factor, with individuals reporting lower levels of depression when they perceived strong support from their social networks (Gabarrell-Pascuet et al., 2023). However, the effectiveness of social support may vary depending on its type and source (Liang et al., 2019; Roskoschinski et al., 2023). This distinction highlights the importance of culturally sensitive interventions that tackle both the emotional and practical dimensions of social support.

Second, our findings revealed that cognitive reappraisal mediates the effect of perceived social support on depression. This indicates that the influence of perceived social support on depression might be partly explained by its impact on emotion regulation processes. Specifically, cognitive reappraisal emerges as a key mediator. This is consistent with the previous view, which highlights interpersonal emotion regulation as a pathway through which social support mitigates depressive symptoms (Marroquín, 2011). Perceived social support may enhance individuals’ ability to utilize adaptive emotion regulation strategies, thereby reducing their vulnerability to depression. Empirical evidence further supports this mediation pathway (Lopez et al., 2024). These findings suggest that social support fosters cognitive reappraisal by providing emotional validation and alternative perspectives, enabling individuals to reinterpret stressors in ways that align with long-term goals (Gross and John, 2003). Our results are consistent with previous studies in the context of Chinese emerging adults (Hua and Ma, 2022). Overall, these results stress the significance of targeting cognitive reappraisal in interventions aimed at reducing depression among emerging adults. By enhancing their ability to regulate emotions effectively, individuals may be better prepared to handle stress and adversity, thus lowering the chances of experiencing depressive symptoms.

Third, our findings suggested that loneliness could mediate the influence of perceived social support on depression. This aligns with studies highlighting the significant role of loneliness in linking social support to depression (Bareket-Bojmel et al., 2021). Loneliness and social isolation were key factors linking mental health stigma to depressive symptoms in young people (Prizeman et al., 2023). Moreover, one recent meta-analysis showed that social support and loneliness are significantly related to symptoms of depression, further emphasizing the mediating role of loneliness (Gabarrell-Pascuet et al., 2023). This is also supported by studies in different populations, such as adolescents and older adults, indicating that the impact of social support on depression is partly due to its effect on loneliness.

In the context of emerging adults, our findings are in line with Liu et al. (2022), who indicated that loneliness was a mediator in the influence of social support on depression among adolescents. Prior research has revealed that social support from friends indirectly affects depression and loneliness in college students (Kulari et al., 2025). Men who have sex with men experience higher levels of depression due to lower social support and higher loneliness, indicating the importance of addressing loneliness in this population (Xie et al., 2024). Social disconnection, often characterized by loneliness, is associated with distress among undergraduate students, further emphasizing loneliness as the mediator in the link between social support and mental health outcomes (Chen et al., 2024). Furthermore, studies across different populations have also revealed the mediating role of loneliness (Liu et al., 2024). These findings suggest that the mediating role of loneliness is not restricted to a particular age group or population but is a common mechanism linking social support to mental health outcomes. This indicates that social support may have a vital effect on reducing loneliness, which subsequently lowers the risk of depression. Thus, our results, along with those of previous studies, highlight that loneliness mediated the impact of perceived social support on depression. This underscores the significance of reducing loneliness in interventions for improving mental health, particularly among emerging adults who are at a vulnerable developmental stage.

Last, our results also indicated the chain-mediated role of cognitive reappraisal and loneliness in the link between perceived social support and depression. Our finding suggests that the impact of social support on depression is not only direct but also indirect, through a sequence of cognitive and emotional processes. Specifically, perceived social support may enhance individuals’ ability to utilize adaptive emotion regulation strategies, like cognitive reappraisal, reducing loneliness and ultimately lowering the risk of depression. This chain-mediated effect is supported by recent research highlighting the interplay among loneliness, emotion regulation, and mental health (Patrichi et al., 2024; Chen et al., 2024). These studies emphasize the significance of adaptive emotion regulation in buffering the negative impact of loneliness on mental health. Further evidence for this chain mediation comes from research on high-risk populations (Valenti et al., 2024). These findings suggest that loneliness acts as a critical mediator, linking emotional and social challenges to mental health. In the context of Chinese emerging adults, this chain mediation may be particularly salient due to cultural expectations of emotional restraint and interdependence (Liang et al., 2019; Liu et al., 2022). These findings underscore the importance of addressing both cognitive and emotional factors in interventions aimed at reducing depression among emerging adults.

This research has three theoretical implications. First, it supplements the existing literature by focusing on emerging adults. By investigating young people in this life stage, the study expands the comprehension of the mediating effect of cognitive reappraisal in the impact of perceived social support on depressive symptoms, with particular emphasis on the impact of cognitive reappraisal. Second, this research offers new evidence that further emphasizes the significance of taking emotion regulation strategies into account as mediating factors in the impact of perceived social support on depression among emerging adults. Last, this study further expands the comprehension of the mediating effect of loneliness in the impact of perceived social support on depression, underscoring the impact of loneliness.

In addition, this research also has some practical implications. First, the results emphasize the significance of targeting cognitive reappraisal in interventions aimed at reducing depression among emerging adults. By developing programs that enhance these skills, mental health professionals and educators can help young people better manage their emotional responses to stress and improve their overall psychological well-being. Second, the study highlights the critical role of loneliness in exacerbating depression among emerging adults. This suggests that efforts to reduce loneliness should be a key component of mental health initiatives. Practical measures could include fostering social connections through group activities, peer support programs, and online platforms that encourage interaction and community building. Third, the findings indicate that improving perceived social support can have a positive influence on mental health outcomes. This underscores the need for supportive networks and resources for emerging adults. Overall, the practical implications of this research suggest a multifaceted approach to addressing depression among emerging adults.

### 4.1 Research limitations

While the present research provides valuable insights, it is important to acknowledge several limitations that need consideration. First, as a cross-sectional study, this research captures a snapshot of the variables at a single point in time, which limits our ability to infer causality. While the findings provide valuable insights into the associations between perceived social support and depression, the nature of the study design means that we cannot definitively establish cause-and-effect relationships. Future research would benefit from longitudinal or other approaches to further explore these relationships and address the limitations inherent in cross-sectional designs. Second, the study focuses on Chinese emerging adults, and the sampling method employed has certain limitations. This limits the generalizability to other populations or cultural contexts. Future studies should consider more varied and representative populations to enhance the applicability of the results. Third, in exploring the relationship between perceived social support and depression, this study focuses on emotion regulation strategies and loneliness as mediating factors. However, the mechanisms underlying this link are not limited to these two variables. Future research should explore the potential contributions of these and other psychological variables to offer a better comprehension of the link between social support and depression.

## 5 Conclusion

This research investigated how perceived social support affects depressive symptoms in Chinese emerging adults. Perceived social support, cognitive reappraisal, and loneliness function as intermediary factors that explain depression levels in Chinese emerging adults. Using cognitive reappraisal more often helps emerging adults in two ways: it directly mediates the link between perceived social support and depression and indirectly influences the link through a chain-mediation model of cognitive reappraisal and loneliness. Thus, cognitive reappraisal contributes to the buffering effect of perceived social support on depression among Chinese emerging adults. In addition, this research revealed that loneliness is a mediator in the link between perceived social support and depression, which expands the complex relationship between loneliness and depression. In a word, our results provided insight into the link between perceived social support and depression and broadened our comprehension of how to utilize adaptive emotion strategies to create more effective interventions that promote mental health and well-being in this vulnerable population.

## Data Availability

The raw data supporting the conclusions of this article will be made available by the authors, without undue reservation.
